# Selective modulation of gene expression in activated normal human peripheral blood mononuclear cells by store-operated calcium entry blocker BTP2

**DOI:** 10.21203/rs.3.rs-2618144/v1

**Published:** 2023-03-13

**Authors:** Divya Shankaranarayanan, Madhav Mantri, Mila Lagman, Carol Li, Vijay K. Sharma, Thangamani Muthukumar, Jenny Z. Xiang, Iwijn De Vlaminck, Khaled Machaca, Manikkam Suthanthiran

**Affiliations:** Division of Nephrology and Hypertension, Department of Medicine, NewYork-Presbyterian-Weill Cornell Medicine; Nancy E. and Peter C. Meinig School of Biomedical Engineering, Cornell University; Division of Nephrology and Hypertension, Department of Medicine, NewYork-Presbyterian-Weill Cornell Medicine; Division of Nephrology and Hypertension, Department of Medicine, NewYork-Presbyterian-Weill Cornell Medicine; Division of Nephrology and Hypertension, Department of Medicine, NewYork-Presbyterian-Weill Cornell Medicine; Division of Nephrology and Hypertension, Department of Medicine, NewYork-Presbyterian-Weill Cornell Medicine; Genomics Resources Core Facility, Department of Microbiology and Immunology, Weill Cornell Medical College; Nancy E. and Peter C. Meinig School of Biomedical Engineering, Cornell University; Department of Physiology and Biophysics, Weill Cornell Medicine; Division of Nephrology and Hypertension, Department of Medicine, NewYork-Presbyterian-Weill Cornell Medicine

## Abstract

Calcium is a critical signaling molecule in many cell types including immune cells. The calcium-release activated calcium channels (CRAC) responsible for store-operated calcium entry (SOCE) in immune cells are gated by STIM family members functioning as sensors of Ca^2+^ store content in the endoplasmic reticulum. We investigated the effect of SOCE blocker BTP2 on human peripheral blood mononuclear cells (PBMC) stimulated with the mitogen phytohemagglutinin (PHA). We performed RNA sequencing (RNA-seq) to query gene expression at the whole transcriptome level and identified genes differentially expressed between PBMC activated with PHA and PBMC activated with PHA in the presence of BTP2. Among the differentially expressed genes, we prioritized genes encoding immunoregulatory proteins for validation using preamplification enhanced real time quantitative PCR assays. We performed multiparameter flow cytometry and validated by single cell analysis that BTP2 inhibits cell surface expression CD25 at the protein level. BTP2 reduced significantly PHA-induced increase in the abundance of mRNAs encoding proinflammatory proteins. Surprisingly, BTP2 did not reduce significantly PHA-induced increase in the abundance of mRNAs encoding anti-inflammatory proteins. Collectively, the molecular signature elicited by BTP2 in activated normal human PBMC appears to be tipped towards tolerance and away from inflammation.

## Introduction

Calcium (Ca^2+^) is a critical signaling molecule in many cell types including T cells and cytosolic Ca^2+^ oscillations impact gene expression[1, 2]. We previously found that the increase in intracellular Ca^2+^ in activated normal human T cells is biphasic; an immediate increase when T cells are signaled via the CD3/T cell receptor complex, and a slower and sustained increase when the T cells are signaled via the costimulatory CD2 protein[3,4]. Importantly, the biphasic Ca^2+^ mobilization was essential for the induction of transcription factors, gene expression, and proliferation of normal human T cells signaled with a synergistic combination of Anti-CD3E monoclonal antibody (mAb) and anti-CD2 mAb[3, 4]. It is established that the immediate increase in cytosolic Ca^2+^ concentration is mediated by the activation of phospholipase C, phosphatidylinositol hydrolysis, generation of inositol triphosphate 3 (IP_3_), and IP_3_ binding to its receptor on the endoplasmic reticulum (ER) and triggering the release of Ca^2+^ from the ER into the cytosol[5-8]. Depletion of Ca^2+^ from the ER stores induces the stromal interaction family members (STIM) to undergo conformational changes and cluster at the ER and plasma membrane (PM) junctions where they recruit members of the Orai1 channel family by diffusional trapping and gating them open to mediate Store Operated Calcium Entry (SOCE)[8-12]. Ca^2+^ influx through SOCE activates calcineurin, a serine-threonine phosphatase that dephosphorylates the transcription factor NFAT-1 leading to its nuclear localization and transcription of genes for T cell activation and function[13]. Emerging data also suggests that SOCE plays a signaling role in NF-kB activation[14].

A nonredundant role for the CRAC channels in-vivo is suggested by the observations that mutations in the CRAC channels are associated with both immunodeficiency and autoimmunity[15-18]. Using a floxed STIM1 hypomorph mouse model, we identified that a functional deficiency of STIM1 is associated with reduced SOCE, impaired NFAT-1 activation, and reduced inflammation[19]. In our study, a cell permeable pyrazole derivative BTP2, a potent inhibitor of CRAC channels and SOCE[20, 21], mimicked several of the effects of STIM1 deficiency in the genetic mouse model.

Existing data and emerging knowledge inspired the current study investigating the effect of BTP2 on the activation of normal human peripheral blood mononuclear cells (PBMC). We performed RNA sequencing (RNA-seq) to examine the effect of BTP2 on genome-wide gene expression in PBMC signaled with PHA. We prioritized and validated differential gene expression of a select panel of mRNAs using a preamplification enhanced real-time quantitative PCR (RT-qPCR) assay developed in our laboratory[22].

Considering the positive signaling role of cytosolic Ca^2+^, we anticipated that the blockade of SOCE with BTP2 would result in the inhibition of PHA-induced gene expression. Indeed, we found that BTP2 blocked activation induced higher expression of multiple genes including genes encoding proinflammatory proteins. Very surprisingly, we found that BTP2 did not inhibit the activation-dependent increase in the expression of mRNAs encoding anti-inflammatory proteins such as immunosuppressive cytokines TGFB1 and IL10, and negative regulators of immunity such as FOXP3 and CTLA4. The newly identified differential effects of BTP2 on activation induced gene expression patterns in normal human PBMC form the basis for this report.

## Results

### RNA-seq of human PBMC.

We isolated PBMC from peripheral venous blood collected from healthy volunteers and incubated PBMC without PHA or BTP2 (control PBMC), with BTP2, with PHA, or with PHA + BTP2 for 16 hours at 37°C in 95% air and 5% CO_2_ atmosphere. Because DMSO was used as the solvent for BTP2, DMSO was also added to control PBMC and PBMC stimulated with PHA.

**Figure 1:**
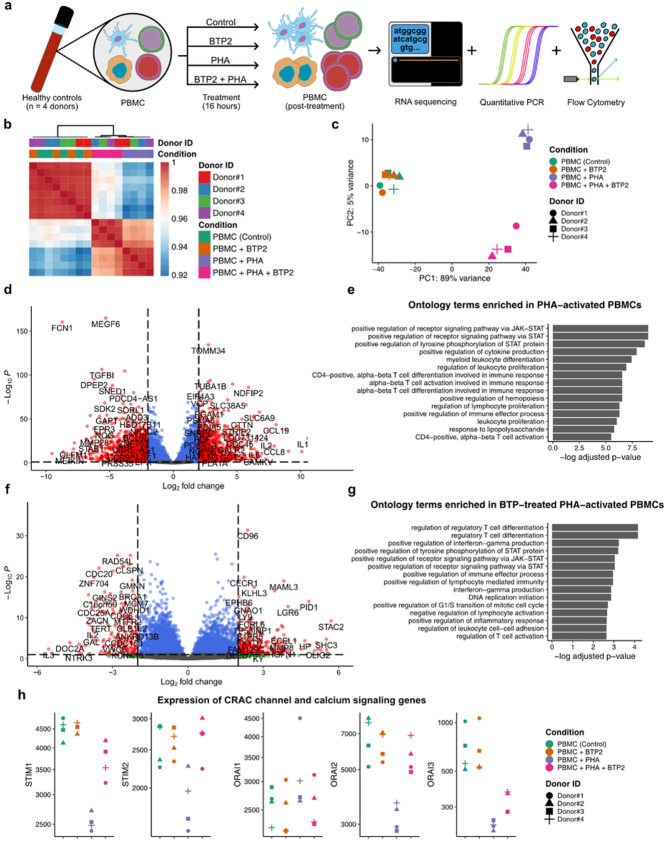
RNA-seq of normal human PBMC activated with PHA in the presence or absence of BTP2. **(a)** Treatment conditions and experimental workflow for profiling normal human PBMC. **(b)** Heatmap showing the pairwise Pearson correlation of gene expression among PBMC from four healthy volunteers.( **c)** Principal component analysis (PCA) projection of RNA-seq data for PBMC from four healthy volunteers across four treatment conditions. **(d)** Volcano plot showing differentially expressed genes (two-sided Wald test, −log2 fold-change < −2.0 or −log2 fold-change > 2.0, and p-value < 0.01) between PBMC + PHA vs. PBMC incubated alone (control) for 16 hours. Dotted lines show the thresholds for significantly enriched genes shown in red. **(e)** Top 15 Gene Ontology (GO) terms for genes enriched in PBMC incubated with PHA as compared to PBMC control. **(f)** Volcano plot showing differentially expressed genes (two-sided Wald test, −log2 fold-change < −2.0 or −log2 fold-change > 2.0 and p-value < 0.01) between PBMC incubated with PHA+ BTP2 vs. PBMC + PHA. Dotted lines show the thresholds for significantly enriched genes shown in red. **(g)** Top 15 Gene Ontology (GO) terms for genes enriched in PBMC + PHA + BTP2 vs. PBMC + PHA. **(h)** Jitter plots showing the log-normalized counts for CRAC channel mRNAs across all four experimental conditions from all four healthy volunteers. Supplementary Table S1 lists base mean counts of all genes from RNA-seq data and log2 fold changes based on comparisons of gene expression in PBMC stimulated with PHA vs. gene expression in control PBMC and adjusted P values. Supplementary Table S2 lists similar parameters based on comparisons of gene counts in PBMC + PHA + BTP2 vs. gene counts PBMC + PHA.

We isolated total RNA from the PBMC and performed RNA-seq to identify gene expression patterns at the whole transcriptome level (Fig. 1a). Prior to differential gene expression analysis, RNA-seq data was processed to obtain the gene expression count data for individual samples (Supplementary Fig. 1a and 1b). These counts were then normalized to total counts, corrected for batch effects, and used for hierarchical clustering based on the Pearson correlation between samples (Supplementary Fig. 1c).

Cluster analysis of RNA sequencing data revealed three distinct groups of samples. PBMC incubated alone and PBMC incubated with BTP2 formed one cluster, PBMC incubated with PHA alone formed another cluster, and the third cluster was formed by PBMC incubated with both PHA and BTP2 (Fig. 1b). Notably, samples obtained from the four different healthy volunteers formed clusters based on experimental conditions and there were no clusters based on individual volunteers indicating that the source of PBMC has a minimal, if any, impact. We employed Principal Component Analysis (PCA) as a means of dimensionality reduction and visualized the data on the first two principal components. PC1 explained 89% of the variance and PC2 5% of the variance, and we observed three groups consistent with the hierarchical clustering results (Fig. 1c). It is evident that PCA analysis shows clear separation based on experimental conditions and not based on individual healthy volunteers. PBMC incubated alone (control) and PBMC incubated with BTP2 showed minimal variation and clustered together suggesting that BTP2 alone has minimal impact on unstimulated PBMC. On the other hand, PBMC stimulated with PHA clustered separately from resting PBMC and from PBMC activated with PHA in the presence of BTP2.

### Differential gene expression analysis.

To comprehensively identify gene expression changes induced by PHA, we compared PHA-treated PBMC to control PBMC using differential gene expression analysis (DGEA). DGEA revealed a significant increase in the expression of 2883 genes (two-tailed Wald test, log fold-change > 1.0 and adjusted p-value < 0.01) and a significant decrease in the expression of 3172 genes following PHA treatment (two-sided Wald test, log fold-change < −1.0 and adjusted p-value < 0.01) (Fig. 1d). Analysis of the DGEA results revealed that the largest increase in expression was observed for genes involved in T cell proliferation, T and B cell differentiation, and genes encoding cytokines and chemokines involved in chemoattraction of T and B cells. A gene ontology enrichment analysis on the panel of 81 genes most upregulated (two-sided Wald test, log2 fold-change > 5.0 and adjusted p-value < 0.01) in PHA-activated PBMC revealed an enrichment of ontology terms related to the positive regulation of receptor signaling pathway via the JAK-STAT pathway, the STAT pathway including positive regulation of tyrosine phosphorylation of STAT protein, positive regulation of cytokine expression, myeloid leukocyte differentiation, and alpha-beta T cell activation and differentiation in immune responses (Fig. 1e).

Supplementary Table S1 lists base mean counts of all genes from RNA-seq data and log2 fold changes based on comparisons of gene expression in PBMC stimulated with PHA vs. gene expression in control PBMC, and adjusted P values. All mRNAs specified in the Results section are highlighted yellow in Supplementary Table S1.

To identify the impact of BTP2 on PBMC activation with PHA, we performed DGEA of PBMC incubated with PHA + BTP2 and PBMC incubated with PHA. DGEA revealed that the impact of BTP2 on PHA-induced gene expression was not monotonic and that both a significant increase in the expression of 745 genes (two-tailed Wald test, log2 fold-change > 1.0 and adjusted p-value < 0.01) and a significant decrease in the expression of 723 genes in the PBMC treated with both PHA and BTP2 (two-sided Wald test, log2 fold-change < −1.0 and adjusted p-value < 0.01) were identified (Fig. 1f). Analysis of the DGEA results revealed that out of 2883 genes which were significantly upregulated in PHA activated cells and 623 genes were significantly downregulated in cells treated with both PHA and BTP2 (two-sided Wald test, log fold-change < −1.0 and adjusted p-value < 0.01). The downregulated genes include the genes involved in T cell proliferation and differentiation such as IL2 and IL2RA (CD25). A gene ontology enrichment analysis on the genes most upregulated (log fold-change > 5.0) in PHA-activated cells but downregulated (log fold-change < −1.0) on BTP2 treatment of PHA-activated PBMC revealed an enrichment of ontology terms related to the regulation of regulatory T cell differentiation and down regulation of interferon gamma and JAK-STAT signaling pathways upregulated in PBMC stimulated with PHA (Fig. 1g). Collectively, these results suggest that BTP2 can positively impact regulatory T cell differentiation and suppress T cell proliferation. Importantly, our analysis identified that the impact of BTP2 on gene expression in stimulated PBMC is not unidirectional, and an almost equal number of genes were upregulated and downregulated by BTP2 in PBMC signaled with PHA.

Supplementary Table S2 lists base mean counts of all genes from RNA-seq data and log2 fold changes based on comparisons of gene expression in PBMC stimulated with PHA + BTP2 vs. gene expression in PBMC + PHA, and adjusted P values. All mRNAs specified in the Results section are highlighted yellow in Supplementary Table S2.

### Impact of BTP2 on mRNAs encoding CRAC channel proteins.

We investigated the effect of PHA on the expression of mRNAs encoding CRAC proteins in PBMC and the impact of BTP2 on PHA-induced alterations in mRNA counts (Fig. 1h).

PHA activation of PBMC decreased the relative counts of STIM1 mRNA (log2fc= −0.82, Adjusted P = 2.06E-17) (Supplementary Table S1) and BTP2 reversed PHA-induced decrease (log2fc= 0.55, Adjusted P = 1.76E-7) (Supplementary Table S2). STIM2 mRNA count was also reduced by PHA activation (log2fc= −0.46, Adjusted P = 0.004) (Supplementary Table S1) and reversed by BTP2 (log2fc = 0.53, Adjusted P = 0.002) (Supplementary Table S2). Statistical analysis showed no significant effect on ORAI1 mRNA counts by either PHA activation (log2fc = 0.29, Adjusted P = 0.173) (Supplementary Table S1) or by the addition of BTP2 to PHA activated PBMC (log2fc= −0.30, Adjusted P = 0.24) (Supplementary Table S2). In contrast, significant reductions in the counts of ORAI2 mRNA (log2fc= −1.06, Adjusted P = 3.56E-09) (Supplementary Table S1) and ORAI3 mRNA (log2fc −1.56, Adjusted P = 2.78E-09) (Supplementary Table S1) were identified with PHA activation and BTP2 significantly reversed PHA-induced decrease in ORAI2 mRNA count (log2fc 0.82, Adjusted P = 4.16E-05) (Supplementary Table S2) and partially reversed PHA-induced decrease in ORAI3 mRNA count (log2fc = 0.49, Adjusted P = 0.15) (Supplementary Table S2). Altogether, we identified that BTP2 reverses PHA-induced decrease in the expression of mRNAs for STIM1, STIM2, ORAI2, and ORAI3 proteins but neither PHA nor BTP2 has a significant effect on the expression of mRNA for ORAI1.

MEGF6 mRNA encodes the calcium ion binding multiple EGF like domains 6 protein, and PHA-activation significantly reduced its expression (log2fc =−5.31, Adjusted P = 1.35E-165) (Supplementary Table S1) and BTP2 significantly reversed the down regulation (log2fc = 1.54, Adjusted P = 3.49E-13) (Supplementary Table S2).

### Impact of BTP2 on mRNAs for T cell surface proteins.

**Figure 2:**
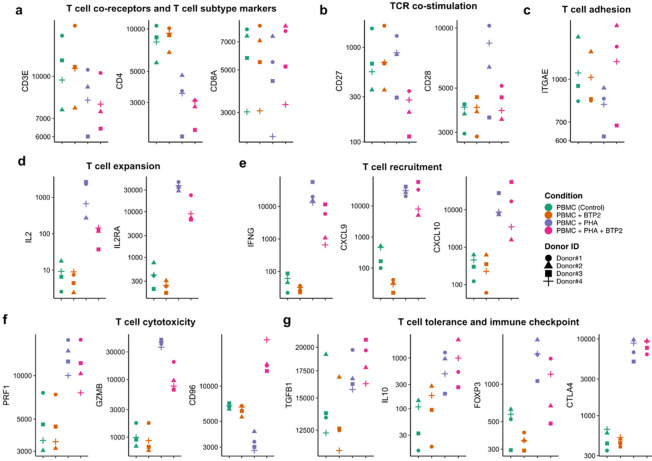
Differential effects of BTP2 on PHA-induced alterations in the expression of mRNAs encoding proteins involved in T cells expansion, recruitment, activation, effector function, and tolerance. **(a-g)** Jitter plots showing the log-normalized counts across the four treatment conditions. **(a)** Pan-T cell surface antigen receptor complex CD3E, CD4 expressed on T helper cells and responsible for recognition of antigenic peptide in the context of HLA class II antigens and CD8 expressed on cytotoxic T cells and responsible for recognition of antigenic peptide in the context of HLA class I antigens; (**b)** T cell co-stimulation receptors CD27 and CD28; (**c)** T cell adhesion marker: surface integrin ITGAE (CD103); (**d)** T cell growth factor IL2 and its receptor IL2RA responsible for T cell clonal expansion; (**e)** IFNG induced T cell recruitment/homing proteins: Chemoattractant molecules CXCL9 and CXCL10; (**f)** T cell cytotoxic proteins PRF1 and GZMB and an antagonist of cytolytic activity, CD96. (**g)** Immunosuppressive cytokines TGFB1 and IL10, Treg cell marker FOXP3 and the master negative regulator of immunity CTLA4.

We analyzed the expression level of CD3E mRNA and found similar levels across all four experimental conditions (Fig. 2a, Supplementary Tables S1 and S2). CD4 mRNA expression was decreased with PHA stimulation of PBMC (log2fc=−1.238, Adjusted P = 9.65E-06) (Fig. 2A, Supplementary Table S1) and was further decreased, albeit non-significantly, by BTP2 (log2fc=−0.309, Adjusted P = 0.438) (Fig. 2a, Supplementary Table S2). CD8A mRNA levels were not significantly different across all four experimental conditions (Fig. 2a, Supplementary Tables S1 and S2).

We compared the expression levels of mRNA encoding co-stimulatory receptors CD27 and CD28 and found differential impacts on these two T cell co-stimulators (Fig. 2b). CD27 mRNA count was not significantly increased by PHA (Supplementary Table S1) but BTP2 reduced the expression level of CD27 mRNA in PHA-stimulated cells (log2fc= −1.82, Adjusted P = 0.003) (Supplementary Table S2). CD28 mRNA count was higher in PBMC + PHA (log2fc = 0.90, Adjusted P = 0.001) (Supplementary Table S1) and BTP2 reduced CD28 mRNA expression (log2fc=−0.753, Adjusted P = 0.021) (Supplementary Table S2). ITGAE (CD103) mRNA count was non-significantly lower following activation of PBMC with PHA and BTP2 partially reversed PHA-induced down regulation (Fig. 2c, Supplementary Tables S1 and S2).

To understand the impact of BTP2 on T cell clonal expansion, we compared expression levels of IL2 mRNA and its receptor CD25 coded by gene IL2RA (Fig. 2d). mRNA for IL2 (log2fc = 7.30, Adjusted P = 3.76E-27) (Supplementary Table S1) and mRNA for IL2RA (log2fc = 6.32, Adjusted P = 3.72E-54) (Supplementary Table S1) were increased by PHA and BTP2 reduced PHA-induced hyper expression of IL2 (log2fc= −3.798, Adjusted P = 5.81E-08) (Supplementary Table S2) and IL2RA (log2fc=−1.658, Adjusted P = 0.0003) (Supplementary Table S2).

To investigate if BTP2 suppresses the recruitment of cells to an inflammatory site (e.g., a rejecting allograft), we compared the expression of mRNAs for the chemokines CXCL9 and CXCL10 (Fig. 2e). CXCL9 mRNA (log2fc = 6.49, Adjusted P = 1.65E-25) (Supplementary Table S1) and CXCL10 mRNA (log2fc = 4.81, Adjusted P = 6.94E-08) (Supplementary Table S1) were both higher in PBMC treated with PHA. Intriguingly, BTP2 did not inhibit PHA-induced increase in the abundance of CXCL9 mRNA (log2fc=−0.199, Adjusted P = 0.850) (Supplementary Table S2) or the PHA-induced increase in the abundance of CXCL10 mRNA (log2fc = 0.56, Adjusted P = 0.699) (Supplementary Table S2). Both CXCL9 and CXCL10 are induced by IFNG. mRNA for IFNG was induced by PHA (log2fc = 8.84, Adjusted P = 2.84E-38) (Fig. 2e, Supplementary Table S1) and BTP2 decreased PHA- induced increase (log2fc=−2.45, Adjusted P = 0.001) (Fig. 2e and Supplementary Table S2).

We asked if BTP2 suppresses mRNA encoding cytotoxic attack molecules PRF1 and GZMB (Fig. 2f). PHA induced an increase in the expression of PRF1 mRNA (log2fc = 1.46, Adjusted P = 2.11E-05) (Supplementary Table S1) and GZMB mRNA (log2fc = 5.253, Adjusted P = 1.70E-39) (Supplementary Table S1) and BTP2 mediated a non-significant decrease in PRF1 mRNA count (log2fc=−0.201, Adjusted P = 0.716) (Supplementary Table S2) and a significant decrease in GZMB mRNA (log2fc=−1.97, Adjusted P = 1.02E-05) (Supplementary Table S2). CD96 is a negative regulator of cytolytic activity of NK cells and T cells. PHA activation decreased CD96 mRNA (log2fc=−0.99, Adjusted P = 2.84E-07) (Fig. 2f, Supplementary Table S1) and BTP2 increased CD96 mRNA count (log2fc = 2.38, Adjusted P = 3.80E-32) (Fig. 2f and Supplementary Table S2). Collectively, these findings suggest that BTP2 would impair the cytolytic activities of T cells and NK cells.

TGFB1 is a potent immunosuppressive cytokine and the abundance of TGFB1 mRNA was not significantly different among the 4 experimental conditions (Fig. 2g) (Supplementary Tables S1 and S2). IL10 mRNA expression was higher in PBMC + PHA vs. PBMC (log2fc = 2.86, Adjusted P = 0.0005) (Supplementary Table S1) and BTP2 did not reduce PHA-induced increased expression of IL10 mRNA (log2fc = 0.48, Adjusted P = 0.738) (Fig. 2g, Supplementary Table S2). The expression of mRNA for the regulatory T cells specification factor FOXP3 was higher in PBMC stimulated with PHA vs. PBMC alone (log2fc = 1.67, Adjusted P = 3.32E-06) (Fig. 2G, Supplementary Table S1) and BTP2 did not significantly decrease activation dependent increase in FOXP3 mRNA (log2fc=−0.79, Adjusted P = 0.071 (Fig. 2g and Supplementary Table S2). We investigated the effect of BTP2 treatment on the expression of mRNA for CTLA4 cell surface protein, a potent negative regulator of immune response. PHA induced a significant increase in CTLA4 mRNA (log2fc = 3.87, Adjusted P = 2.38E-56) (Fig. 2g, Supplementary Table S1) and BTP did not reduce the PHA-induced increase in CTLA4 mRNA count (log2fc = 0.11, Adjusted P = 0.782) (Fig. 2g, Supplementary Table S2).

Altogether, RNA-seq of PBMC identified that BTP2 treatment significantly suppresses mRNA encoding proteins responsible for T cell expansion, activation, and cytotoxicity without significantly reducing the expression of mRNA encoding negative regulators of immunity.

### Validation of differential gene expression using preamplification enhanced RT-qPCR assays.

We used an orthogonal platform, RT-qPCR assay developed in our laboratory[22] to measure absolute copy numbers of transcripts; we prioritized for measurement mRNAs encoding immunoregulatory proteins implicated in autoimmunity and organ transplantation[23, 24]. Table 1 shows absolute copy numbers of mRNAs measured in four consecutive experiments, the median and 25th and 75th percentile values, multiple group comparison P values calculated using Kruskal Wallis test, and pairwise comparisons P values calculated using Mann Whitney U tests.

The abundance of 18S rRNA is relatively stable across cell types and experimental conditions and therefore was used as a housekeeping (reference) gene in our study. As shown in Table 1, 18S rRNA copy numbers were not significantly different across the four experimental conditions (P = 0.82, Kruskal Wallis multiple comparison test). Pairwise comparisons of PBMC vs. PBMC + BTP2 (P = 0.89, Mann Whitney test), PBMC vs. PBMC + PHA (P = 0.89), and PBMC + PHA vs. PBMC + PHA + BTP2 (P = 0.51) showed no significant differences in the abundance of 18S rRNA across the 4 experimental conditions. The lack of a significant difference in the abundance of 18S rRNA copy number suggests that significant differences observed with a mRNA of interest are due to differences in expression due to the experimental condition rather than due to technical artifacts.

### mRNAs for T cell surface proteins.

Measurement of absolute copy number of mRNAs using the RT-qPCR assays showed excellent agreement with the alterations in mRNA counts identified by RNA-seq. As found by RNA-seq, expression levels of CD3E mRNA or CD8A mRNA did not vary across all four experimental conditions (Table 1). CD4 mRNA copy number was numerically lower following PHA activation and further reduced by BTP2 (Table 1).

As found by RNA-seq, BTP2 reduced the PHA-induced increase in CD27 mRNA copy number; BTP2 also reduced the PHA-induced increase in CD28 mRNA copy number (Table 1). RT-qPCR assays results were also concordant with RNA-seq data regarding CD103 mRNA - a non-significant decrease following activation with PHA and partial reversal by BTP2 (Table 1).

### mRNAs for IL2 and IL2RA (CD25).

As observed with RNA-seq, PHA-activation of PBMC caused a significant increase in IL2 mRNA copy number and BTP2 significantly reduced PHA-induced increase (Table 1). PHA-activation of PBMC mediated a significant increase in IL2RA copy number and BTP2 reduced significantly PHA-induced increase (Table 1).

### mRNAs for IFNG, CXCL9 and CXCL10 and CXCR3.

As found by RNA-seq, PHA-induced a significant increase in IFNG mRNA copy number and BTP2 partially reduced IFNG mRNA copy number (Table 1). Measurement of absolute copy numbers of CXCL9 mRNA and CXCL10 mRNA using RT-qPCR assays validated both the increase in the abundance of CXCL9 mRNA and CXCL10 mRNA following PHA activation, and the lack of inhibition by BTP2 of the PHA induced increase in the abundance of CXCL9 mRNA or CXCL10 mRNA (Table 1).

CXCR3 is a receptor for both CXCL9 and CXCL10 and its abundance was not altered in PBMC by PHA stimulation or by BTP2 (Table 1).

### mRNAs for PRF1, GZMB and CD96.

RT-qPCR assay measurements of PRF1 and GZMB copy numbers validated RNA-seq findings that PHA stimulation increases PRF1 mRNA copy number and GZMB mRNA copy number in PBMC and BTP2 mediates a non-significant decrease in PRF1 mRNA copy number and a significant decrease in GZMB mRNA copy number (Table 1).

### mRNAs for TGFB1, IL10, FOXP3 and CTLA4.

Measurement of mRNAs encoding immunosuppressive cytokines TGFB1 and IL10 and the negative immune regulators FOXP3 and CTLA4 confirmed the RNA-seq findings that BTP2 does not significantly reduce the expression of mRNAs encoding these anti-inflammatory mediators. PHA increased the abundance of mRNAs for TGFB1(P = 0.06) and IL10 (P = 0.03) in PBMC and BTP2 did not reduce the PHA-induced increase in TGFB1 mRNA (P > 0.99) or the increase in IL10 mRNA (P = 0.69) (Table 1). PHA also increased the abundance of mRNAs for FOXP3 (P = 0.03) and CTLA4 (P = 0.03) and BTP2 did not reduce the PHA-induced increase in FOXP3 mRNA (P = 0.11) or the increase in CTLA4 mRNA(P = 0.20) (Table 1).

### Single cell analysis for cell surface expression of IL2 receptor alpha (CD25).

**Figure 3:**
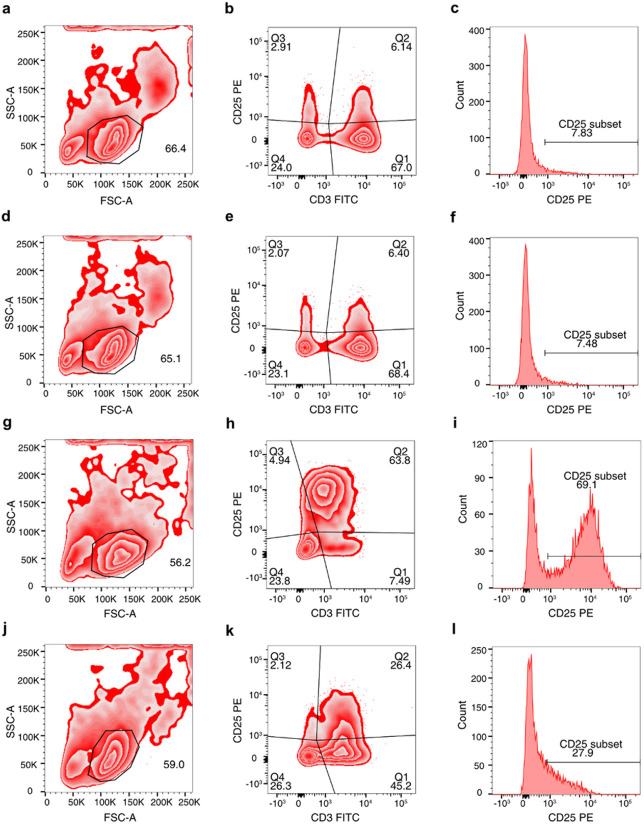
Inhibition of cell surface expression of IL2 receptor alpha (CD25) by BTP2. Peripheral blood mononuclear cells were incubated either alone (control), with 1000 nM BTP2, with PHA (2μg/ml), or with PHA (2μg/ml) + BTP2 (1000nM) for 16 hours at 37°C in 95% air and 5% CO_2_ atmosphere. At the end of incubation, the cells were washed and labeled with FITC mouse IgG2a isotype control mAb and PE IgG1 isotype control mouse mAb or FITC IgG2a mouse anti-human CD3 mAb and PE IgG1 mouse anti-human CD25 mAb. Flow cytometry data was acquired on a FACSCanto II using FACSDiva software (8.0.1) and the FCS files were analyzed using FlowJo 10.8.1 software. Fig. 3a, d, g, and j are contour plots based on cell size (forward scatter) and granularity (side scatter) of control PBMC (Fig. 3a), PBMC treated with BTP2 (Fig. 3d), PBMC+ PHA (Fig. 3G) or PBMC+PHA+BTP2 (Fig. 3j). Each contour plot shows the gates and the percentage of cells analyzed. Fig. 3b, f, h, and k are bivariate plots of gated PBMC from all four experimental and the cells segregated based on FITC CD3E florescence signal and PE CD25 florescence signal. The vertical line in the bivariate plots is based on corresponding isotype mAb labeling discriminating CD3E+ cells from CD3E− cells and the horizontal line is based on corresponding isotype mAb discriminating CD25+ cells from CD25− cells. Fig. 3p,f, i, and I are single color histograms of CD25+ cells from all four experiments conditions. The horizontal line indicates the percentage of CD25+ cells.

We performed multiparameter flow cytometry analysis to determine the effect of BTP2 on PHA-induced cell surface expression of IL2RA (CD25). Figure 3 is representative flow cytometry data from all four experimental conditions and shows that PHA increases the percentage of CD25 + cells and BTP2 decreases cell surface expression of CD25.

Results from four consecutive experiments are shown in Table 2. Table 2a shows data from each of the four experiments and Table 2b is a summary of statistics from the four consecutive experiments. Pairwise comparisons showed that PHA stimulation significantly increases CD25 display on the cell surface and BTP2 significantly decreases the cell surface expression of CD25 (Table 2b). An analogous pattern of an increase with PHA and a decrease with BTP2 was also observed with the MCF of CD25 positive cells (Table 2b). Altogether, multiparameter flow cytometry analysis of CD25 expression at the protein level confirmed and extended our findings that BTP2 inhibits PHA-induced expression of CD25 at the pre-translational level.

## Discussion

We identified that the SOCE blocker BTP2 has a gene specific effect on PHA-induced alterations in the abundance of mRNAs in normal human PBMC. Intriguingly, the activation induced increases in the abundance of mRNAs encoding anti-inflammatory proteins were not reduced significantly by BTP2 whereas activation induced increases in the abundance of mRNAs encoding proinflammatory proteins were significantly reduced.

**Figure 4:**
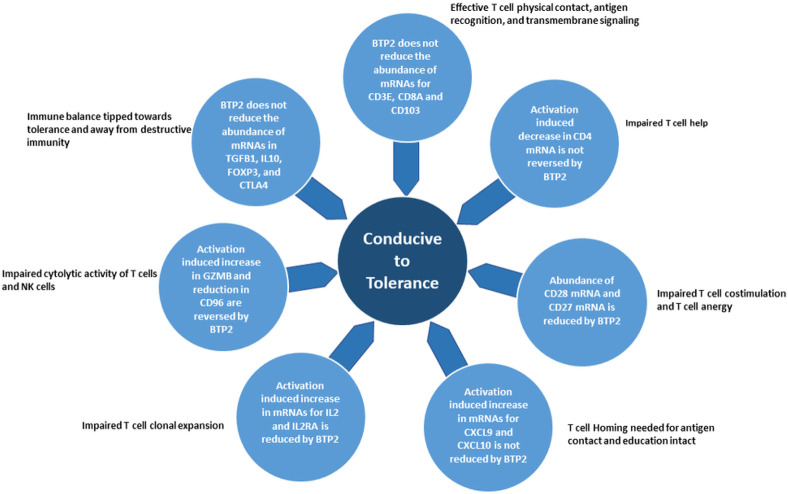
Potential consequences of BTP2 mediated selective modulation of gene expression in normal human PBMC. The lack of inhibitory effect of BTP2 on the expression of mRNAs for CD3E, CD8A, and CD103 should be permissive of physical contact, antigen recognition, and T cell signaling. BTP2 does not reverse activation induced decrease in mRNA for CD4 and this could lead to impaired helper cell function including T follicular helper cell function required for antibody production. Down regulation of mRNA for CD27 and CD28 by BTP2 should impair T cell co-stimulation and promote T cell anergy. T cell homing needed for antigen contact and education should proceed unhindered since BTP2 does not reduce activation induced up regulation of CXCL9 mRNA and CXCL10 mRNA. BTP2 mediated inhibition of activation induced mRNA for IL2 and mRNA for IL2RA is predicted to prevent T cell clonal expansion, a prerequisite for destructive immunity. BTP2 mediated reduction in mRNA for GZMB and the increase in CD96 are expected to impair cytolytic activity of T cells and NK cells. The lack of reduction by BTP2 of mRNAs for the immunosuppressive cytokines TGFB1 and IL10, and the lack of reduction by BTP2 of activation induced increase in mRNAs for the negative regulators of immunity, FOXP3 and CTLA4, are postulated to promotes tolerance. Collectively, BTP2 mediated selective modulation of gene expression in normal human PBMC is hypothesized to tip the immune balance towards tolerance and away from inflammation.

The immune profile elicited by BTP2 appears conducive to antigen specific tolerance (Fig. 4). The finding that BTP2 does not alter the expression of mRNAs for T cell surface molecules CD3E, CD8A, and CD103 suggests that T cell antigen education via physical contact with the cognate antigen and transmembrane signaling should proceed uninterrupted. On the other hand, the expected outcomes of BTP-associated reduction in the mRNAs for CD4, CD27, and CD28 include impaired T cell helper function, sub-optimum T cell co-stimulation, and T cell anergy. CXCR3 mRNA and mRNAs for the chemokines CXCL9 and CXCL10 were not reduced by BTP2, and a predicted outcome is unhindered trafficking of antigen reactive cells to their target cells. Such homing is unlikely to have an adverse consequence since neither clonal expansion nor cytolytic activity would be realized since BTP2 reduced mRNAs for T cell growth factor IL2 and its receptor IL2RA and mRNA for the cytolytic effector granzyme B. In striking contrast, mRNA for TGFB1, a potent immunosuppressive cytokine, and mRNA for the cytokine synthesis inhibitory factor IL10 were not reduced by BTP2. Moreover, mRNA for FOXP3, a specification factor T regulatory cells, and mRNA for the negative regulator CTLA4 were not reduced by BTP2. Collectively, the molecular signature elicited by BTP2 in activated normal human PBMC appears to be tipped towards tolerance and away from inflammation.

Several features of our research design enabled the identification of selective modulation of gene expression by BTP2. First, we used normal human PBMC as indicator cells rather than the malignant Jurkat CD4 + T cells or T cell clones to assess the effects of BTP2. The PBMC activation model is also physiological with respect to T cell co-stimulation. PHA was used to signal PBMC in our study and PHA is considered a superior stimulator of lymphocytes compared to several other mitogens including the widely used lectin concanavalin A[25, 26]. Importantly, we performed unbiassed RNA-seq to query gene expression at the whole transcriptome level and identified differential gene expression in an unbiased fashion. We prioritized mRNAs encoding proteins implicated in autoimmunity, allograft rejection, and allograft tolerance for validation using an orthogonal platform and confirmed differential expression by absolute quantification of mRNA copy number. The measurement of absolute copy number of mRNAs in the RT-qPCR assays[22] obviated the inherent limitations of relative quantification of mRNAs in RT-qPCR assays.

The CD3 proteins are an integral component of the T cell receptor (TCR) antigen recognition complex and the CD4 and CD8 proteins are expressed by helper T cells and cytotoxic T cells, respectively[27-29]. BTP2 had a minimal impact on mRNAs encoding CD3E or CD8A. On the other hand, BTP2 reduced mRNAs for CD4, CD27, and CD28 in PBMC. Full T cell activation is dependent upon co-stimulatory signals and the T cell CD27 and CD28 are prominent T cell co-stimulatory proteins[30, 31]. An expected consequence of BTP2 reducing their expression is impaired T cell co-stimulation and T cell anergy[32]. Altogether, the T cell profile elicited by BTP2 is permissive of T cell antigen recognition and transmembrane signaling, impaired T cell helper function, and defective T cell co-stimulation and anergic T cells (Fig. 4).

IL2 is the primary trophic factor for T cell clonal expansion and the acquisition of IL2RA by activated T cells is necessary for the formation of high-affinity IL2R trimeric complex[33, 34]. BTP2 reduced activation induced increased expression of mRNAs for IL2 and IL2RA. A predicted outcome is impaired T cell clonal expansion (Fig. 4).

IFNG was first identified as secretory product of PHA-stimulated leucocytes and is a prototypic proinflammatory cytokine[35]. BTP2 reduced activation induced increase in IFNG mRNA in PBMC. CXCL9 and CXCL10 are IFNG induced chemokines that signal T cells via CXCR3 displayed their cell surface[36, 37]. PHA mediated a significant increase in the abundance of mRNA for both chemokines and intriguingly, BTP2 did not reduce significantly the induced higher abundance. These surprising findings and the additional observation that BTP2 does not affect CXCR3 mRNA expression indicate that cellular traffic to target sites such as an allograft will not be jeopardized by BTP2 and antigen-experienced cells, a prerequisite for tolerance induction, could emerge (Fig. 4).

Perforin is a pore forming protein and granzymes enter cells via the pores in the plasma membrane and mediate cell death by apoptosis. PHA-activation significantly increased the expression levels of both perforin and granzyme B. heightened intragraft expression of perforin and granzymes have been repeatedly identified during an episode of acute allograft rejection[23, 34]. Thus, BTP2 mediated decrease in GZMB mRNA is likely to benefit the transplanted organ. We found that the abundance of mRNA for CD96, expressed on the surface of T cells and NK cells and a negative regulator NK/T cell cytolytic activity[38, 39] is increased by BTP2. This data suggests that BTP2 would help constrain cytolytic effector mechanisms implicated in autoimmunity and allograft rejection (Fig. 4).

Genome wide profiling using RNA-seq identifying that inhibition of SOCE results in diminished expression of genes in activated PBMC is predictable considering the positive signaling role of SOCE in the activation of immune cells. The unanticipated finding from our investigation is that genes encoding anti-inflammatory proteins were largely unaffected by BTP2. In this study, the abundance of mRNAs encoding potent immunosuppressive cytokines TGFB1 and IL10 were not reduced significantly by BTP2. Furthermore, BTP2 did not reduce significantly the activation induced increase in mRNA for FOXP3 protein, a specification factor for regulatory T cells, and activation induced increase in mRNA for CTLA4, a master negative regulator of immunity[40,41]. Intragraft over-expression of FOXP3 + regulatory cells is a feature of tolerant allografts[42,43] and CTLA4 mRNA is over expressed in tolerant kidney allograft recipients[44] (Fig. 4).

Our investigation has limitations. BTP2 has effects on TRPM4, TRPC3, and TRPC5 channels[21] in addition to CRAC channels, and some of our results might be related to the effect of BTP2 on these other channels. We have not deciphered whether the differential effect of BTP2 on gene expression is cell autonomous[45] or due to differential sensitivity of cell subtypes. Real time simultaneous imaging of calcium in the ER lumen, cytosol, and mitochondria[45] and single cell RNA-seq may help resolve this issue. It is important to investigate whether our findings are translatable to in-vivo settings. BTP2 (also known as YM58483) has been reported to inhibit T cell responses in-vivo[46] and is efficacious as an anti-inflammatory agent[47-50]. The BTP2 associated tolerogenic signature identified in this study suggest a mechanistic basis for the efficacy observed in these preclinical models. Additional studies including transplantation across strong histocompatibility barriers are needed to evaluate the utility of BTP2 in the transplantation setting.

In conclusion, BTP2 has differential effects on activation induced gene expression in normal human PBMC signaled with PHA. Intriguingly, BTP2 did not significantly reduce the abundance of mRNAs encoding anti-inflammatory proteins whereas the mRNAs encoding proinflammatory proteins were significantly reduced by this pyrazole derivative. The tipping of the immune repertoire towards tolerance and away from inflammation profile by BTP2 may be of value for the management of autoimmune disease states and for protecting lifesaving organ transplants.

## Methods

### Reagents.

Ficoll-Paque^™^ Premium solution was purchased from GE Healthcare Bio-Science AB, Pittsburgh, PA. Phytohemagglutinin was purchased from Thermo Fisher Scientific, Fair Lawn, NJ. BTP2 was purchased from EMD Millipore Corporation, Burlington, MA. Dimethyl sulfoxide (DMSO) was purchased from Sigma Aldrich, Saint Louis, MO. Dulbecco’s Phosphate Buffered Saline (PBS) and heat inactivated fetal bovine serum (FBS) were purchased from GIBCO, Grand Island, NY. Mouse FITC IgG2a mAb, mouse PE IgG1 mAb, FITC IgG2a mouse anti-human CD3 mAb, and PE IgG1 mouse anti-human CD25 mAb were purchased from BD Biosciences, San Jose, CA.

### Isolation of normal human PBMC.

Peripheral venous whole blood was obtained from healthy volunteers after obtaining informed written consent. The research project # 1208012870 was approved by the Weill Cornell Medicine Institutional Review Board. The authors confirm that all research was performed in accordance with relevant guidelines/regulations, and the research involving human research was performed in accordance with the Declaration of Helsinki. PBMC were isolated by density gradient centrifugation using Ficoll-Paque^™^ centrifugation and suspended in complete medium composed of RPMI 1640 + L-Glutamine + HEPES (25mM), Penicillin (100 units/ml), and Streptomycin (0.1 mg/ml) and 5% FBS (complete medium). PBMC were washed twice with PBS and the final wash performed with complete medium. PBMC at 1x10^6^/ml were incubated without PHA or BTP2 (control), with BTP2 (1000 nM), with PHA (2μg/ml), or with PHA (2μg/ml) + BTP2 (1000 nM) for 16 hours at 37°C in 95% air and 5% CO_2_ atmosphere. Because DMSO was used to dissolve BTP2, DMSO was added to control PBMC and to PBMC stimulated with PHA. After overnight incubation, PBMC were retrieved and separate aliquots of PBMC suspended in complete medium aliquots were used for total RNA isolation and single cell analysis by flow cytometry.

### Isolation of total RNA from PBMC.

PBMCs were washed with PBS (1000 μl) and centrifuged at 13,400 g for 2 minutes in room temperature. The supernatant was discarded, and RNAlater (50 μl) and Buffer RLT with β-mercaptoethanol (350 μl) were added to the cell pellet. The pellet was mechanically lysed with a 1ml 25G 5/8 syringe. The lysate was transferred to a QIAshredder spin column and centrifuged at maximum speed for 2 minutes at room temperature. Total RNA was isolated from the lysate using RNeasy mini kit, according to the manufacturer’s protocol (Qiagen). The quantity (absorbance at 260nm) and purity (ratio of the absorbance at 260 and 280 nm) of the total RNA isolated from the lysate were measured using the NanoDrop^®^ ND-1000 UV-Vis spectrophotometer (Thermo Scientific).

### RNA library preparation for RNA-seq.

The total RNA samples were treated with DNase using RNase-Free DNase kit (Qiagen) according to the manufacturer’s protocol to remove DNA prior to RNA sample library preparation and sequencing. The RNA library preparation and RNA sequencing were performed by the Genomics Core Laboratory at Weill Cornell Medicine. The RNA libraries were prepared using the TruSeq RNA Library Prep Kit (Illumina) and sequenced using NovaSeq 6000 System (Illumina) as 100 x 2 paired-end reads.

### Preprocessing of RNA-seq data.

Raw data from RNA sequencing experiments was aligned to the human genome (Assembly: GRCh37/hg19) using STAR short read aligner software (v-2.6)[51] for individual samples. The gene expression counts for individual samples were estimated by setting the --quantMode argument to GeneCounts. The gene expression values for individual samples were compiled into a single table for downstream analysis.

### Analysis of RNA-seq data.

Count matrix containing gene expression counts and the corresponding metadata and alignment statistics for all samples were loaded into R (v-4.0.3). The matrix was converted into a DESeqDataSet object for downstream analysis using the DESeq2 package (v-1.30.1)[52]. SizeFactors and Dispersion values were calculated for individual genes using the DESeq2 package and genes with less than ten copies across all samples were removed from the analysis. The data was normalized using variance stabilizing and rlog transformations (VST) method implemented in the DESeq2 package. The normalized data was batch-corrected across the samples using linear models implemented in the limma package (v-3.46.0) [53]. The batch corrected data was transferred back into the DESeqDataSet object and the control PBMC sample was set as the reference. Pairwise correlations were calculated for individual samples and visualized on a Heatmap. Dimensional reduction was performed on batch corrected data using Principal Component Analysis, which was visualized using the DESeq2 package. Differential Gene Expression Analysis (DGEA) was performed using the DESeq2 method. Effect size shrinkage was used on DGEA results using apeglm (Approximate posterior estimation for GLM coefficients) method[54] implemented in lfcShrink function in the DESeq2 package. The DGEA results containing log fold-changes and adjusted p-values were exported in tables and visualized on volcano plots.

### Preamplification enhanced real time quantitative polymerase chain reaction (RT-qPCR) assay.

Total RNA isolated from the PBMC was reverse transcribed (RT) to cDNA using the TaqMan reverse transcription kit (Applied Biosystems) at the concentration of 1.0 μg of total RNA in 100 μl volume, as previously described[22]. The reverse transcription reaction contained TaqMan reverse transcription buffer (1x), 500 μM each of 4 dNTPs, Random Hexamer (2.5 μM), RNase inhibitor (0.4 Unit/μl), MultiScribe Reverse Transcriptase (1.25 Unit/μl), and Magnesium Chloride (5.5mM). The RT mixture was incubated at 25oC for 10 min, 48oC for 30 min, and 95oC for 5 min.

We designed gene specific oligonucleotide primers and TaqMan fluorogenic probes and measured a custom panel of mRNAs and 18S rRNA using pre-amplification enhanced real-time quantitative PCR (RT-qPCR) assays. Supplementary Table S5 lists the oligonucleotide primers and gene specific TaqMan probes used to measure absolute copy number of transcripts.

The RT-qPCR assay consisted of two sequential steps[22]. In the first step, the cDNA was pre-amplified with gene-specific primer pairs in a final reaction volume of 10.0 μl in a 0.2 ml PCR tube and each sample contained cDNA (3.0 μl), Platinum^®^ Multiplex PCR Master Mix (5.0 μl), 1.68 μl primer mix (50 μM sense, 50 μM antisense primer per gene), and water (0.32 μl) to a final volume of 10 μl. PCR amplification was set up in a Veriti thermal cycler (Applied Biosystems) and the PCR profile consisted of an initial hold at 95oC for 2 min, 11 cycles of denaturing at 95oC for 30 seconds, primer annealing at 60oC for 90 seconds and extension at 72oC for 1 min, and final extension at 72oC for 10 min and final hold at 4oC. Following 11 cycles of amplification, we diluted the PCR amplicons by adding 290 μl of TE buffer to the 10 μl PCR reaction. In the second step, absolute copy number of mRNAs were quantified using the Bak standard curve in the RT-qPCR assay on the Applied Biosystems QuantStudio^™^ 6 Real-Time PCR system (ThermoFisher) using diluted PCR amplicons (2.5 μl), TaqMan^™^ Fast Universal PCR Master Mix (2X), no AmpErase^™^ UNG (Applied Biosystems), sense and antisense primers (50 μM each), TaqMan^™^ probes (100 μM), and water (7.1 μl) in a final reaction volume of 20 μl. We established the Bak standard curve with 6 log_10_ concentrations of Bak amplicon (starting copy number 2.5 x 10^6^ million copies of Bak mRNA) as the template and PCR amplification with Bak-specific primer pair and detection using Bak-specific TaqMan probe. The amplification efficiencies of Bak and the mRNAs quantified in this study were greater than 90% in the RT-qPCR assays. Absolute copy number of mRNA was reported as mRNA copies per microgram of total RNA.

### Flow cytometry analysis of PBMC.

PBMC, 1x10^6^ cells/ml of RPMI 1640 + 5% heat inactivated fetal bovine serum were incubated without PHA or BTP2 (control), with BTP2 (1000 nM), with PHA (2 μg/ml), or with PHA (2 μg/ml) + BTP2 (1000 nM) for 16 hours at 37°C in 95% air and 5% CO_2_ atmosphere. At the end of incubation, the cells were washed and labeled with FITC mouse IgG2a,k isotype control mAb (G155-178, BD Pharmingen) and PE Mouse IgG1, k isotype control mAb (MOPC-21, BD Pharmingen) or FITC mouse IgG2a, k anti-human CD3E mAb (HIT3a, BD Pharmingen) and PE mouse IgG1, k anti-human CD25 mAb (M-A251, BD Pharmingen). Each isotype mAb (10 μl) and each antibody marker (10 μl) for CD3E and CD25 was added to 100 μl of cell suspension in 5ml falcon tube. The sample tubes were vortexed and incubated in dark at 4 C for 30 min in refrigerator. After incubation, cells were washed by adding 3 ml of cold working wash buffer (PBS containing 1% FBS and 0.1% FBS and 0.1% wt/vol sodium azide). Cells were then resuspended in 400 μL of working fixative reagent (PBS + 0.1% sodium azide +1% fetal bovine serum + 37% formalin) and flow cytometry analysis was performed. Flow cytometry data was acquired on a FACSCanto II (BD Biosciences) using FACSDiva software (8.0.1) and the FCS files were analyzed using FlowJo 10.8.1 software (Beckton Dickinson and Company).

## Figures and Tables

**Table 1:** Validation of differentially expressed mRNAs using preamplification enhanced RT-qPCR assays.

Gene	Treatment Condition	Experiment No.				Median	IQ25	IQ75	P	P (Pairwise comparison)		
		1	2	3	4					PBMC vs PBMC + BTP2	PBMC vs PBMC + PHA	PBMC + PHA vs PBMC + PHA + BTP2
18S	PBMC	5.9E + 09	2.3E + 10	3.6E + 10	2.9E + 10	2.6E + 10	1.9E + 10	3.1E + 10	0.82	0.89	0.89	0.51
	PBMC + BTP2	4.2E + 09	3.3E + 10	3.4E + 10	3.1E + 10	3.2E + 10	2.4E + 10	3.3E + 10				
	PBMC + PHA	1.5E + 10	2.6E + 10	3.1E + 10	1.8E + 10	2.2E + 10	1.7E + 10	2.7E + 10				
	PBMC + PHA + BTP2	2.3E + 10	2.3E + 10	3.6E + 10	2.6E + 10	2.5E + 10	2.3E + 10	2.9E + 10				
CD3E	PBMC	5.1E + 05	4.7E + 05	1.1E + 06	8.2E + 05	6.6E + 05	5.0E + 05	8.9E + 05	0.83	0.89	0.69	0.89
	PBMC + BTP2	3.4E + 05	9.0E + 05	6.7E + 05	7.3E + 05	7.0E + 05	5.9E + 05	7.8E + 05				
	PBMC + PHA	4.9E + 05	1.1E + 06	7.7E + 05	4.5E + 05	6.3E + 05	4.8E + 05	8.4E + 05				
	PBMC + PHA + BTP2	5.5E + 05	4.7E + 05	5.7E + 05	5.1E + 05	5.3E + 05	5.0E + 05	5.5E + 05				
CD4	PBMC	1.0E + 05	7.8E + 04	2.1E + 05	1.4E + 05	1.2E + 05	9.6E + 04	1.6E + 05	0.003	> 0.99	0.11	0.06
	PBMC + BTP2	6.1E + 04	1.8E + 05	1.2E + 05	1.5E + 05	1.4E + 05	1.1E + 05	1.6E + 05				
	PBMC + PHA	5.4E + 04	1.3E + 05	5.0E + 04	4.3E + 04	5.2E + 04	4.8E + 04	7.3E + 04				
	PBMC + PHA + BTP2	4.7E + 04	3.7E + 04	3.3E + 04	4.1E + 04	3.9E + 04	3.6E + 04	4.3E + 04				
CD8A	PBMC	2.1E + 05	2.2E + 05	3.3E + 05	1.4E + 05	2.1E + 05	1.9E + 05	2.5E + 05	0.95	0.69	>0.99	0.63
	PBMC + BTP2	1.6E + 05	4.0E + 05	1.8E + 05	1.3E + 05	1.7E + 05	1.5E + 05	2.4E + 05				
	PBMC + PHA	1.9E + 05	4.0E + 05	2.2E + 05	7.8E + 04	2.0E + 05	1.6E + 05	2.7E + 05				
	PBMC + PHA + BTP2	3.2E + 05	3.2E + 05	2.4E + 05	1.2E + 05	2.8E + 05	2.1E + 05	3.2E + 05				
CD27	PBMC	9.6E + 04	4.0E + 04	1.2E + 05	8.8E + 04	9.2E + 04	7.6E + 04	1.0E + 05	0.0002	0.34	0.11	0.03
	PBMC + BTP2	7.0E + 04	7.2E + 04	5.9E + 04	8.0E + 04	7.1E + 04	6.7E + 04	7.4E + 04				
	PBMC + PHA	1.4E + 05	2.2E + 05	1.9E + 05	9.5E + 04	1.6E + 05	1.3E + 05	2.0E + 05				
	PBMC + PHA + BTP2	3.5E + 04	2.5E + 04	1.0E + 04	2.3E + 04	2.4E + 04	2.0E + 04	2.7E + 04				
CD28	PBMC	2.3E + 04	2.4E + 04	5.8E + 04	2.9E + 04	2.6E + 04	2.4E + 04	3.6E + 04	0.02	0.89	0.03	0.03
	PBMC + BTP2	1.6E + 04	4.9E + 04	3.0E + 04	2.9E + 04	2.9E + 04	2.6E + 04	3.4E + 04				
	PBMC + PHA	8.7E + 04	1.1E + 05	6.6E + 04	6.6E + 04	7.7E + 04	6.6E + 04	9.2E + 04				
	PBMC + PHA + BTP2	4.4E + 04	2.6E + 04	4.4E + 04	2.7E + 04	3.5E + 04	2.6E + 04	4.4E + 04				
CD103 (ITGAE)	PBMC	1.4E + 03	4.6E + 03	6.5E + 03	3.2E + 03	3.9E + 03	2.7E + 03	5.1E + 03	0.13	0.89	0.06	0.11
	PBMC + BTP2	1.1E + 03	7.1E + 03	1.5E + 03	3.2E + 03	2.4E + 03	1.4E + 03	4.1E + 03				
	PBMC + PHA	6.7E + 02	2.2E + 03	5.9E + 02	8.9E + 02	7.8E + 02	6.5E + 02	1.2E + 03				
	PBMC + PHA + BTP2	3.7E + 03	4.9E + 03	7.7E + 02	2.5E + 03	3.1E + 03	2.1E + 03	4.0E + 03				
IL2	PBMC	2.5E + 02	2.7E + 02	5.3E + 02	3.1E + 02	2.9E + 02	2.6E + 02	3.7E + 02	< 0.0001	0.89	0.03	0.03
	PBMC + BTP2	1.6E + 02	3.4E + 02	4.0E + 02	3.4E + 02	3.4E + 02	2.9E + 02	3.5E + 02				
	PBMC + PHA	1.7E + 05	1.5E + 04	1.2E + 05	3.5E + 04	7.9E + 04	3.0E + 04	1.4E + 05				
	PBMC + PHA + BTP2	1.1E + 04	3.7E + 03	1.9E + 03	6.5E + 03	5.1E + 03	3.2E + 03	7.5E + 03				
IL2RA	PBMC	2.5E + 04	2.9E + 04	3.7E + 04	3.8E + 04	3.3E + 04	2.8E + 04	3.8E + 04	< 0.0001	0.03	0.03	0.03
	PBMC + BTP2	6.6E + 03	2.2E + 04	9.9E + 03	2.4E + 04	1.6E + 04	9.1E + 03	2.2E + 04				
	PBMC + PHA	4.1E + 06	4.9E + 06	5.4E + 06	4.2E + 06	4.5E + 06	4.1E + 06	5.1E + 06				
	PBMC + PHA + BTP2	1.7E + 06	5.4E + 05	8.2E + 05	1.0E + 06	9.3E + 05	7.5E + 05	1.2E + 06				
IFNG	PBMC	2.4E + 02	2.1E + 02	5.3E + 02	5.1E + 02	3.7E + 02	2.3E + 02	5.1E + 02	0.0001	0.34	0.03	0.11
	PBMC + BTP2	1.6E + 02	2.0E + 02	3.3E + 02	2.6E + 02	2.3E + 02	1.9E + 02	2.8E + 02				
	PBMC + PHA	5.0E + 05	2.9E + 05	8.5E + 05	2.4E + 05	3.9E + 05	2.7E + 05	5.9E + 05				
	PBMC + PHA + BTP2	1.9E + 05	1.3E + 04	3.3E + 05	1.1E + 04	1.0E + 05	1.3E + 04	2.3E + 05				
CXCL9	PBMC	1.7E + 04	1.8E + 04	2.3E + 04	1.4E + 04	1.8E + 04	1.6E + 04	1.9E + 04	< 0.0001	0.03	0.03	> 0.99
	PBMC + BTP2	1.1E + 03	1.3E + 03	2.7E + 02	3.1E + 02	6.9E + 02	3.0E + 02	1.1E + 03				
	PBMC + PHA	1.7E + 06	1.7E + 06	2.8E + 06	1.2E + 06	1.7E + 06	1.5E + 06	2.0E + 06				
	PBMC + PHA + BTP2	2.9E + 06	2.3E + 05	4.1E + 06	3.6E + 05	1.7E + 06	3.3E + 05	3.2E + 06				
CXCL10	PBMC	3.4E + 03	1.2E + 04	1.8E + 04	1.5E + 04	1.3E + 04	9.5E + 03	1.6E + 04	0.0009	0.69	0.03	0.89
	PBMC + BTP2	5.3E + 02	1.6E + 04	1.3E + 04	6.1E + 03	9.6E + 03	4.7E + 03	1.4E + 04				
	PBMC + PHA	7.8E + 04	2.2E + 05	1.3E + 06	3.1E + 05	2.7E + 05	1.8E + 05	5.5E + 05				
	PBMC + PHA + BTP2	2.7E + 05	3.2E + 04	2.9E + 06	1.4E + 05	2.1E + 05	1.1E + 05	9.4E + 05				
CXCR3	PBMC	3.5E + 04	1.2E + 05	1.7E + 05	6.8E + 04	9.4E + 04	5.9E + 04	1.3E + 05	0.78	0.89	0.69	0.49
	PBMC + BTP2	2.7E + 04	2.3E + 05	1.2E + 05	6.6E + 04	9.1E + 04	5.6E + 04	1.5E + 05				
	PBMC + PHA	6.6E + 04	2.7E + 05	1.5E + 05	8.5E + 04	1.1E + 05	8.0E + 04	1.8E + 05				
	PBMC + PHA + BTP2	6.7E + 04	9.4E + 04	8.7E + 04	4.7E + 04	7.7E + 04	6.2E + 04	8.9E + 04				
PRF1	PBMC	1.8E + 05	6.3E + 04	1.8E + 05	1.7E + 05	1.8E + 05	1.5E + 05	1.8E + 05	0.0007	0.69	0.03	0.49
	PBMC + BTP2	1.2E + 05	1.2E + 05	1.2E + 05	1.8E + 05	1.2E + 05	1.2E + 05	1.4E + 05				
	PBMC + PHA	5.9E + 05	8.7E + 05	6.7E + 05	4.4E + 05	6.3E + 05	5.5E + 05	7.2E + 05				
	PBMC + PHA + BTP2	7.9E + 05	4.0E + 05	6.1E + 05	3.9E + 05	5.0E + 05	3.9E + 05	6.6E + 05				
GZMB	PBMC	8.3E + 04	2.8E + 04	5.9E + 04	4.8E + 04	5.3E + 04	4.3E + 04	6.5E + 04	< 0.0001	0.34	0.03	0.03
	PBMC + BTP2	5.9E + 04	4.5E + 04	2.7E + 04	3.8E + 04	4.1E + 04	3.5E + 04	4.9E + 04				
	PBMC + PHA	2.2E + 06	3.1E + 06	4.0E + 06	1.6E + 06	2.7E + 06	2.1E + 06	3.3E + 06				
	PBMC + PHA + BTP2	1.3E + 06	4.5E + 05	3.3E + 05	2.9E + 05	3.9E + 05	3.2E + 05	6.7E + 05				
TGFB1	PBMC	2.7E + 05	2.8E + 05	3.9E + 05	2.3E + 05	2.7E + 05	2.6E + 05	3.1E + 05	0.04	0.49	0.06	>0.99
	PBMC +	1.9E	5.1E	2.7E	2.2E	2.4E +	2.1E	3.3E				
	BTP2	+ 05	+ 05	+ 05	+ 05	05	+ 05	+ 05				
	PBMC + PHA	5.0E + 05	8.2E + 05	5.6E + 05	3.0E + 05	5.3E + 05	4.5E + 05	6.3E + 05				
PBMC + PHA + BTP2	7.0E + 05	4.3E + 05	6.4E + 05	3.4E + 05	5.4E + 05	4.1E + 05	6.6E + 05
IL10	PBMC	2.2E + 03	7.9E + 03	8.8E + 03	9.6E + 03	8.4E + 03	6.5E + 03	9.0E + 03	0.0009	0.89	0.03	0.69
	PBMC + BTP2	1.3E + 03	2.5E + 04	6.0E + 03	1.2E + 04	9.2E + 03	4.8E + 03	1.6E + 04				
	PBMC + PHA	1.7E + 05	1.4E + 05	1.2E + 05	5.2E + 04	1.3E + 05	1.0E + 05	1.5E + 05				
	PBMC + PHA + BTP2	6.9E + 04	1.7E + 05	2.5E + 04	8.1E + 04	7.5E + 04	5.8E + 04	1.0E + 05				
FOXP3	PBMC	1.4E + 04	8.2E + 03	8.0E + 03	7.8E + 03	8.1E + 03	7.9E + 03	9.7E + 03	0.0012	0.69	0.03	0.11
	PBMC + BTP2	3.7E + 03	8.7E + 03	4.7E + 03	9.2E + 03	6.7E + 03	4.4E + 03	8.9E + 03				
	PBMC + PHA	4.4E + 04	8.1E + 04	2.5E + 04	4.5E + 04	4.4E + 04	3.9E + 04	5.4E + 04				
	PBMC + PHA + BTP2	3.3E + 04	1.3E + 04	1.2E + 04	2.7E + 04	2.0E + 04	1.3E + 04	2.9E + 04				
CTLA4	PBMC	2.9E + 03	1.0E + 04	1.5E + 04	1.4E + 04	1.2E + 04	8.2E + 03	1.4E + 04	0.0004	0.89	0.03	0.20
	PBMC + BTP2	2.1E + 03	1.8E + 04	7.1E + 03	1.2E + 04	9.3E + 03	5.9E + 03	1.3E + 04				
	PBMC + PHA	3.4E + 04	2.9E + 05	1.7E + 05	1.8E + 05	1.8E + 05	1.4E + 05	2.1E + 05				
	PBMC + PHA + BTP2	3.0E + 04	1.4E + 05	1.4E + 05	1.3E + 05	1.3E + 05	1.0E + 05	1.4E + 05				

Peripheral blood mononuclear cells (PBMC, 1x10^6^ cells/ml of RPMI 1640+ 5% heat inactivated fetal bovine serum) were incubated without BTP2 or PHA (PBMC), with 1000 nM BTP2 (PBMC + BTP2), with 2 μg/ml PHA (PBMC + PHA), or with 1000 nM BTP2 + 2 μg/ml PHA (PBMC + PHA + BTP2) for 16 hours at 37°C in 95% air and 5% CO_2_ atmosphere. At the end of incubation, total RNA was isolated from PBMC, reverse transcribed to cDNA and absolute copy number of mRNA was quantified using the preamplification enhanced RT-qPCR assay. The sequence and location of the oligonucleotide primers and gene specific TaqMan probes designed at the Weill Cornell Medicine Gene Expression Monitoring Core, NY to quantify transcripts are listed in Supplementary Table S5. Absolute copy number of 18S rRNA per microgram of total RNA and absolute copy number of mRNA per microgram of total RNA from each experimental condition and from the four consecutive experiments are shown along with the median and 25^th^ and 75^th^ percentile copy numbers.

† P values calculated using Kruskal-Wallis test of no differences in mRNA copy number (dependent variable) among the PBMC incubated without BTP2 or PHA, PBMC incubated with 1000 nM BTP2, PBMC incubated with 2 μg/ml PHA or PBMC incubated with 1000 nM BTP2+ 2 μg/ml PHA groups.

‡ P values calculated using Mann-Whitney test of no difference between two groups.

**Table 2a:** Inhibition of PHA-induced cell surface expression of IL2 receptor alpha (CD25) by BTP2: Individual experiments*

Experiment	PBMC		PBMC + BTP2		PBMC + PHA		PBMC + PHA + BTP2	
	Percentage	MCF	Percentage	MCF	Percentage	MCF	Percentage	MCF
Exp. 1	6.82	1633	7.54	1591	55.9	7629	31.4	2865
Exp. 2	7.83	1639	7.48	1402	69.1	7397	27.9	1728
Exp. 3	6.81	1652	7.57	1606	68.2	4316	21.6	1588
Exp. 4	8.8	1501	8.28	1636	78.2	7834	36.2	2400

**Table 2b:** Inhibition of PHA-induced cell surface expression of IL2 receptor alpha (CD25) by BTP2: Four consecutive experiments*

PBMC	PBMC + BTP2	PBMC + PHA	PBMC + PHA + BTP2	P Value^†^		P Value^‡^ PBMC Vs.	P Value^‡^ PBMC + BTP2 Vs.	P Value^‡^ PBMC + PHAVs.	P Value^‡^ PBMC + PHA + BTP2 Vs.
(n = 4)	(n = 4)	(n = 4)	(n = 4)						
Percentage of CD25 Positive PBMC Median (25th and 75th percentile)									
7.33	7.56	68.65	29.65	< 0.0001	PBMC	-	-	-	
					PBMC + BTP2	0.89	-	-	0.03
(6.81 – 8.8)	(7.48 – 8.28)	(55.9 – 78.2)	(21.6 – 36.2)		PBMC + PHA	0.03	0.03	-	0.03
					PBMC + PHA + BTP2	0.03	0.03	0.03	-
MCF of CD25 Positive PBMC Median (25th and 75th percentile)									
1636	1599	7513	2064	0.003	PBMC	-	-	-	0.2
					PBMC + BTP2	0.34	-	-	0.2
(1501 – 1652)	(1402 – 1636)	(4316 – 7834)	(1588 – 2865)		PBMC + PHA	0.03	0.03	-	0.03
					PBMC + PHA + BTP2	0.2	0.2	0.03	-

* Peripheral blood mononuclear cells (PBMC, 1x10^6^ cells/ml of RPMI 1640 + 5% heat inactivated fetal bovine serum) were incubated without BTP2 or PHA (PBMC), with 1000 nM BTP2 (PBMC + BTP2), with 2 μg/ml PHA (PBMC + PHA), or with 1000 nM BTP2 + 2 μg/ml PHA (PBMC + PHA + BTP2) for 16 hours at 37°C in 95% air and 5% CO_2_ atmosphere. At the end of incubation, the cells were washed and labeled with FITC-IgG2a isotype control mAb and PE IgG1 isotype control mAb or FITC IgG2a mouse anti-human CD3 mAb and PE IgG1 mouse anti-human CD25 mAb. Flow cytometry data were acquired on a FACSCanto II using FACSDiva software (8.0.1) and the FCS files were analyzed using FlowJo 10.8.1 software. The percentage and the mean channel fluorescence (MCF) of CD25 positive cells from each experiment (Table 2A) and the median (25th and 75th percentile) values from 4 consecutive experiments (Table 2B) are shown.

† P values calculated using Kruskal-Wallis test of no differences in the percentage or MCF of CD25 positive cells (dependent variable) among the PBMC incubated without BTP2 or PHA (PBMC), PBMC incubated with 1000 nM BTP2, PBMC incubated with 2 μg/ml PHA or PBMC incubated with 1000 nM BTP2 + 2 μg/ml PHA groups.

‡ P values calculated using Mann-Whitney test of no difference in the percentage or MCF of CD25 positive cells (dependent variable) between two groups.

## Data Availability

Data reported in this manuscript will be made available upon request. Count matrices containing gene expression counts for all samples have been made available as Supplementary Table S3. Metadata and alignment statistics for all samples have been made available as Supplementary Table S4. Data supporting the findings in this study are included in the main article and associated files. **The RNA sequencing datasets generated and analyzed during the current study are available in the** NCBI’s Gene Expression Omnibus under the accession number GSE226570 (https://www.ncbi.nlm.nih.gov/geo/query/acc.cgi?acc=GSE226570). The secure token to access GSE226570 while it remains in private status is: idadgmigdhidhsb.

## References

[1] Trebak M., Kinet J.P. Calcium signalling in T cells. Nat. Rev. Immunol 19(3), 154–169. DOI: 10.1038/S41577-018-0110-7 (2019). 30622345 PMC6788797

[2] Dolmetsch R.E., Xu K., Lewis R.S. Calcium oscillations increase the efficiency and specificity of gene expression. Nature 392(6679), 933–6. DOI: 10.1038/31960 (1998). 9582075

[3] Suthanthiran M. A novel model for antigen-dependent activation of normal human T cells. Transmembrane signaling by crosslinkage of the CD3/T cell receptor-alpha/beta complex with the cluster determinant 2 antigen. J. Exp. Med. 171(6), 1965–79. DOI: 10.1084/jem.171.6.1965 (1990). 1972176 PMC2187964

[4] Sehajpal P.K., Sharma V.K., Ingulli E., Stenzel K.H., Suthanthiran M. Synergism between the CD3 antigen- and CD2 antigen-derived signals. Exploration at the level of induction of DNA-binding proteins and characterization of the inhibitory activity of cyclosporine. Transplantation 55(5), 1118–24. DOI: 10.1097/00007890-199305000-00035 (1993). 8098881

[5] Berridge M.J., Irvine R.F. Inositol trisphosphate, a novel second messenger in cellular signal transduction. Nature 312(5992), 315–21. DOI: 10.1038/312315a0 (1984). 6095092

[6] Streb H., Irvine R.F., Berridge M.J., Schulz I. Release of Ca2 + from a nonmitochondrial intracellular store in pancreatic acinar cells by inositol-1,4,5-trisphosphate. Nature 306(5938), 67–9. DOI: 10.1038/306067a0 (1983). 6605482

[7] Berridge M.J., Irvine R.F. Inositol phosphates and cell signalling. Nature 341(6239), 197–205. DOI: 10.1038/341197a0 (1989). 2550825

[8] Putney J.W. Jr. Receptor-regulated calcium entry. Pharmacol. Ther. 48(3), 427 – 34. DOI: 10.1016/0163-7258(90)90059-b (1990). 1982181

[9] Liou J. STIM is a Ca2 + sensor essential for Ca2+-store-depletion-triggered Ca2 + influx. Curr. Biol. 15(13), 1235–41. DOI: 10.1016/j.cub.2005.05.055 (2005). 16005298 PMC3186072

[10] Hogan P.G., Lewis R.S., Rao A. Molecular basis of calcium signaling in lymphocytes: STIM and ORAI. Annu. Rev. Immunol. 28, 491–533. DOI: 10.1146/annurev.immunol.021908.132550 (2010). 20307213 PMC2861828

[11] Prakriya M., Lewis R.S. Store-Operated Calcium Channels. Physiol. Rev. 95(4), 1383 – 436. DOI: 10.1152/physrev.00020.2014 (2015). 26400989 PMC4600950

[12] Taylor C.W., Machaca K. IP(3) receptors and store-operated Ca(2+) entry: a license to fill. Curr. Opin. Cell Biol. 57,1–7. DOI: 10.1016/j.ceb.2018.10.001 (2019). 30368032

[13] Macian F. NFAT proteins: key regulators of T-cell development and function. Nat. Rev. Immunol. 5(6), 472–84. DOI: 10.1038/nri1632 (2005). 15928679

[14] Berry C.T., May M.J., Freedman B.D. STIM-and Orai-mediated calcium entry controls NF-kappaB activity and function in lymphocytes. Cell Calcium 74, 131–143. DOI: 10.1016/j.ceca.2018.07.003 (2018). 30048879 PMC6415950

[15] Feske S. A mutation in Orai1 causes immune deficiency by abrogating CRAC channel function. Nature 441(7090), 179–85. DOI: 10.1038/nature04702 (2006). 16582901

[16] Yu F. Novel ORAI1 Mutation Disrupts Channel Trafficking Resulting in Combined Immunodeficiency. J. Clin. Immunol. 41(5), 1004–1015. DOI: 10.1007/s10875-021-01004-8 (2021). 33650027 PMC8249264

[17] Picard C. STIM1 mutation associated with a syndrome of immunodeficiency and autoimmunity. N. Engl. J. Med. 360(19), 1971–80. DOI: 10.1056/NEJMoa0900082 (2009). 19420366 PMC2851618

[18] Vaeth M., Feske S. Ion channelopathies of the immune system. Curr. Opin. Immunol. 52, 39–50. DOI:10.1016/j.coi.2018.03.021 (2018). 29635109 PMC6004246

[19] Yu F. Chronic reduction of store operated Ca(2+) entry is viable therapeutically but is associated with cardiovascular complications. J. Physiol. 600(22), 4827–4848. DOI: 10.1113/JP283811 (2022). 36181482

[20] Djuric S.W. 3,5-Bis(trifluoromethyl)pyrazoles: a novel class of NFAT transcription factor regulator. J. Med. Chem. 43(16), 2975–81. DOI: 10.1021/jm990615a (2000). 10956206

[21] Takezawa R. A pyrazole derivative potently inhibits lymphocyte Ca2 + influx and cytokine production by facilitating transient receptor potential melastatin 4 channel activity. Mol. Pharmacol. 69(4), 1413–20. DOI: 10.1124/mol.105.021154 (2006). 16407466

[22] Muthukumar T. Messenger RNA for FOXP3 in the urine of renal-allograft recipients. N. Engl. J. Med. 353(22),2342–51. DOI: 10.1056/NEJMoa051907 (2005). 16319383

[23] Lubetzky M.L., Salinas T., Schwartz J.E., Suthanthiran M. Urinary Cell mRNA Profiles Predictive of Human Kidney Allograft Status. Clin. J. Am. Soc. Nephrol. 16(10), 1565–1577. DOI: 10.2215/CJN.14010820 (2021). 33906907 PMC8499006

[24] Nissaisorakarn V., Lee J.R., Lubetzky M.L., Suthanthiran M. Urine biomarkers informative of human kidney allograft rejection and tolerance. Hum. Immunol. 79(5), 343–355. DOI: 10.1016/j.humimm.2018.01.006 (2018). 29366869

[25] Miller K. The stimulation of human B and T lymphocytes by various lectins. Immunobiology 165(2), 132 – 46. DOI: 10.1016/S0171-2985(83)80055-2 (1983). 6354918

[26] Beinke C., Port M., Lamkowski A., Abend M. Comparing seven mitogens with PHA-M for improved lymphocyte stimulation in dicentric chromosome analysis for biodosimetry. Radiat. Prot. Dosimetry 168(2), 235–41. DOI: 10.1093/rpd/ncv286 (2016). 25958413 PMC4884885

[27] Reinherz E.L. Antigen recognition by human T lymphocytes is linked to surface expression of the T3 molecular complex. Cell 30(3), 735 – 43. DOI: 10.1016/0092-8674(82)90278-1 (1982). 6982759

[28] Meuer S.C. Clonotypic structures involved in antigen-specific human T cell function. Relationship to the T3 molecular complex. J. Exp. Med. 157(2), 705–19. DOI: 10.1084/jem.157.2.705 (1983). 6185617 PMC2186929

[29] Janeway C.A. Jr. The T cell receptor as a multicomponent signalling machine: CD4/CD8 coreceptors and CD45 in T cell activation. Annu. Rev. Immunol. 10, 645–74. DOI: 10.1146/annurev.iy.10.040192.003241 (1992). 1534242

[30] Mueller D.L., Jenkins M.K., Schwartz R.H. An accessory cell-derived costimulatory signal acts independently of protein kinase C activation to allow T cell proliferation and prevent the induction of unresponsiveness. J. Immunol. 142(8), 2617–28 (1989). 2522963

[31] Jenkins M.K., Taylor P.S., Norton S.D., Urdahl K.B. CD28 delivers a costimulatory signal involved in antigen-specific IL-2 production by human T cells. J. Immunol. 147(8), 2461–6 (1991). 1717561

[32] Appleman L.J., Boussiotis V.A. T cell anergy and costimulation. Immunol. Rev. 192, 161–80. DOI: 10.1034/j.1600-065x.2003.00009.x (2003). 12670403

[33] Smith K.A. Interleukin-2: inception, impact, and implications. Science 240(4856), 1169–76. DOI: 10.1126/science.3131876 (1988). 3131876

[34] Malek T.R., Castro I. Interleukin-2 receptor signaling: at the interface between tolerance and immunity. Immunity 33(2), 153 – 65. DOI: 10.1016/j.immuni.2010.08.004 (2010). 20732639 PMC2946796

[35] Wheelock E.F. Interferon-Like Virus-Inhibitor Induced in Human Leukocytes by Phytohemagglutinin. Science 149(3681), 310–1. DOI: 10.1126/science.149.3681.310 (1965). 17838106

[36] Lira S.A, Furtado G.C. The biology of chemokines and their receptors. Immunol Res 54(1–3), 111 – 20. DOI: 10.1007/s12026-012-8313-7 (2012). 22466932 PMC4115270

[37] Schenk A.D., Rosenblum J.M., Fairchild R.L. Chemokine-directed strategies to attenuate allograft rejection. Clin. Lab Med. 28(3), 441 – 54, vii. DOI: 10.1016/j.cll.2008.07.004 (2008). 19028262 PMC2632546

[38] Georgiev H, Ravens I, Papadogianni G, Bernhardt G. Coming of Age: CD96 Emerges as Modulator of Immune Responses. Front Immunol. 9, 1072. DOI: 10.3389/fimmu.2018.01072 (2018). 29868026 PMC5966540

[39] Mittal D. CD96 Is an Immune Checkpoint That Regulates CD8(+) T-cell Antitumor Function. Cancer Immunol Res 7(4), 559–571. DOI: 10.1158/2326-6066.CIR-18-0637 (2019). 30894377 PMC6445751

[40] Walker L.S. Treg and CTLA-4: two intertwining pathways to immune tolerance. J. Autoimmun 45(100):49–57. DOI: 10.1016/j.jaut.2013.06.006 (2013). 23849743 PMC3989116

[41] Murakami N., Riella L.V. Co-inhibitory pathways and their importance in immune regulation. Transplantation 98(1), 3–14. DOI: 10.1097/TP.0000000000000169 (2014). 24978034

[42] Graca L., Cobbold S.P., Waldmann H. Identification of regulatory T cells in tolerated allografts. J. Exp. Med. 195(12), 1641–6. DOI: 10.1084/jem.20012097 (2002). 12070291 PMC2193557

[43] Kawai T. HLA-mismatched renal transplantation without maintenance immunosuppression. N. Engl. J. Med. 358(4), 353–61. DOI: 10.1056/NEJMoa071074 (2008). 18216355 PMC2819046

[44] Lee J. Tolerant Kidney Transplant Recipients Display a Unique CTLA-4 Dominant Urinary Cell mRNA Signature. Transplantation 106(9S), 44. DOI: 10.1097/01.tp.0000885464.51550.4d (2022).

[45] Courjaret R., Dib M., Machaca K. Spatially restricted subcellular Ca(2+) signaling downstream of store-operated calcium entry encoded by a cortical tunneling mechanism. Sci. Rep. 8(1), 11214. DOI: 10.1038/S41598-018-29562-9 (2018). 30046136 PMC6060099

[46] Ohga K., Takezawa R., Arakida Y., Shimizu Y., Ishikawa J. Characterization of YM-58483/BTP2, a novel store-operated Ca2 + entry blocker, on T cell-mediated immune responses in vivo. Int. Immunopharmacol 8(13–14), 1787–92. DOI: 10.1016/j.intimp.2008.08.016 (2018). 18793756

[47] Zhang W., Qi Z., Wang Y. BTP2, a Store-Operated Calcium Channel Inhibitor, Attenuates Lung Ischemia-Reperfusion Injury in Rats. Inflammation 40(3), 778–787. DOI: 10.1007/s10753-017-0522-8 (2017). 28168659

[48] Qi Z. The Central Analgesic Mechanism of YM-58483 in Attenuating Neuropathic Pain in Rats. Cell Mol Neurobiol. 36(7), 1035–43. DOI: 10.1007/s10571-015-0292-5 (2016). 26514127 PMC11482431

[49] Yoshino T. YM-58483, a selective CRAC channel inhibitor, prevents antigen-induced airway eosinophilia and late phase asthmatic responses via Th2 cytokine inhibition in animal models. Eur. J. Pharmacol. 560, 225–33. DOI: 10.1016/j.ejphar.2007.01.012 (2007). 17307161

[50] Ohga K. The suppressive effects of YM-58483/BTP-2, a store-operated Ca2 + entry blocker, on inflammatory mediator release in vitro and airway responses in vivo. Pulm. Pharmacol Ther. 21, 360–9. DOI: 10.1111/bph.13104 (2008). 17977764

[51] Dobin A STAR: ultrafast universal RNA-seq aligner. Bioinformatics 29(1),15–21. doi: 10.1093/bioinformatics/bts635 (2013) 23104886 PMC3530905

[52] Love M.I., Huber W. & Anders S. Moderated estimation of fold change and dispersion for RNA-seq data with DESeq2. Genome Biol. 15, 550. 10.1186/s13059-014-0550-8 (2014). 25516281 PMC4302049

[53] Ritchie M.E. limma powers differential expression analyses for RNA-sequencing and microarray studies. Nucleic Acids Research 43(7), e47. DOI: 10.1093/nar/gkv007 (2015). 25605792 PMC4402510

[54] Zhu A., Ibrahim J.G., Love M.I.. Heavy-tailed prior distributions for sequence count data: removing the noise and preserving large differences. Bioinformatics 35(12), 2084–2092 DOI: 10.1093/bioinformatics/bty895 (2019). 30395178 PMC6581436

